# Consequences of above-ground invasion by non-native plants into restored vernal pools do not prompt same changes in below-ground processes

**DOI:** 10.1093/aobpla/plab042

**Published:** 2021-07-05

**Authors:** Amber C Churchill, Akasha M Faist

**Affiliations:** 1 Department of Ecology, Evolution and Behavior, University of Minnesota, St. Paul, MN 55108, USA; 2 Department of Animal and Range Sciences, New Mexico State University, Las Cruces, NM 88003, USA

**Keywords:** Invasive litter decomposition, multivariate community analysis, restoration ecology, vernal pools

## Abstract

Given the frequent overlap between biological plant invasion and ecological restoration efforts it is important to investigate their interactions to sustain desirable plant communities and modify long-term legacies both above- and below-ground. To address this relationship, we used natural reference, invaded and created vernal pools in the Central Valley of California to examine potential changes in direct and indirect plant effects on soils associated with biological invasion and active restoration ecosystem disturbances. Our results showed that through a shift in vegetation composition and changes in the plant community tissue chemistry, invasion by non-native plant species has the potential to transform plant inputs to soils in vernal pool systems. In particular, we found that while invasive plant litter decomposition was driven by seasonal and interannual variability, associated with changes in precipitation, the overall decomposition rates for invasive litter was drastically lower than native species. This shift has important implications for long-term alterations in plant-based inputs to soils in an amplifying feedback to nutrient cycling. Moreover, these results were independent of historic active restoration efforts. Despite the consistent shift in plant litter decomposition rates and community composition, we did not detect associated shifts in below-ground function associated with invasion by non-native plants. Instead, soil C:N ratios and microbial biomass did not differ between invaded and naturally occurring reference pools but were reduced in the manipulated created pools independent of invasion levels. Our results suggest that while there is an observed invasive amplifying feedback above-ground this trajectory is not represented below-ground, and restoration legacies dominated 10 years after practices were applied. Restoration practices that limit invasive plant feedbacks and account for soil legacy recovery, therefore offer the best solution for disturbed ephemeral ecosystems.

## Teaser

Applications of restoration practices in wetland systems frequently must contend with plant invasion by non-targeted species, and these invading plants can undermine restoration goals through impacts both above- and below-ground. In this study we examined the impacts of invasive plants among natural and created vernal pools in California and found that invasion had a large effect on the plants present in the community and especially influenced plant litter decomposition rates. These invasive species caused changes can have implications for long-term alterations in plant-based inputs to soils; however, we did not detect associated shifts in below-ground function associated with invasion by non-native plants.

## Introduction

The vegetation community has a key role in influencing soil properties through increased soil physical stabilization (Gray 1974), altering hydrology (Huxman *et al.* 2005; Donohue *et al.* 2012), influencing soil biota (Collins *et al.* 2016; Tamura *et al.* 2017; Zhang *et al.* 2020) and changing nutrient availability (Ehrenfeld 2003; Allison and Vitousek 2004; Chapman *et al.* 2006; Liao *et al.* 2008), among other known features (Eviner and Chapin 2003). In addition to vegetation influencing soil structure and function, soil properties in turn influence successive plant growth thereby causing potential for bidirectional plant–soil feedbacks (Kulmatiski *et al.* 2008; Bardgett and Wardle 2010; Kardol *et al.* 2013; Hobbie 2015). In systems dominated by native species with relatively low disturbance levels, existing feedbacks between plants and soils that have a history of co-occurrence may be relatively static, held in balance through stabilizing factors such as consistent nutrient cycling or accumulation of organic material (Reinhart and Callaway 2006; Hawkes *et al.* 2013; Kardol *et al.* 2013; Van der Putten *et al.* 2013). The introduction of invasive species, however, can shift plant–soil interactions in an amplifying, or positive feedback, trajectory further altering above-ground vegetation composition and successive soil properties (Klironomos 2002; Rodríguez-Echeverría *et al.* 2009; Kardol *et al.* 2013; Mack *et al.* 2019). Positive feedbacks from invasive plant species can therefore shift abiotic and biotic ecosystem properties out of perceived stability further pushing novel trajectories both above-ground and below-ground (Simberloff and Von Holle 1999; Suding *et al.* 2004; Reinhart and Callaway 2006), ultimately jeopardizing ecosystem management goals associated with ecological restoration (Purcell *et al.* 2019).

The impact of changes in plant–soil feedbacks associated with plant invasion on ecosystem processes is varied (Prasse *et al.* 2015; Maietta *et al.* 2020), and includes factors such as establishment order and changes in dispersal for natives and target species (Aronson and Galatowitsch 2008) as well as potential cascading effects on soil processes (Ehrenfeld 2003). In many cases, invasive plants have an increased rate of growth when compared to other community members (Daehler 2003; Leger and Rice 2003; Jakobs *et al.* 2004), which can lead to an increase in litter deposition at the soil surface (Ehrenfeld *et al.* 2005; Mariotte *et al.* 2017). Increased litter layers can enhance soil moisture levels (Wolkovich *et al.* 2009), reduce light availability (Adili *et al.* 2013) and create a physical barrier hindering rare or subdominant species (Mariotte *et al.* 2017; Faist and Beals 2018) thereby generating amplifying ecological feedbacks that support further changes in the ecosystem by their presence. In one strategy, the fast-growing plant material of some invasive species contains higher nitrogen content than non-invasive natives (Vitousek and Walker 1989; Ashton *et al.* 2005; Liao *et al.* 2008), that then introduces higher quality (lower carbon to nitrogen ratios, less lignin) plant litter inputs to soils and facilitates more rapid decomposition (Allison and Vitousek 2004; Spirito *et al.* 2014). In these systems, this cycle of rapid growth and faster decomposition from the invasive species can change the nutrient cycling and soil chemistry (Ehrenfeld 2003; Allison and Vitousek 2004; Liao *et al.* 2008) as well as the biomass of the soil microbial community that utilizes soil nutrients and the accumulation of soil carbon (Klironomos 2002; Ehrenfeld *et al.* 2005; Angeloni *et al.* 2006; Fickbohm and Zhu 2006; de la Peña *et al.* 2016). Changes in soil properties caused by invasive plants can therefore have a diverse and continued array of effects on ecosystem processes over time (Ehrenfeld *et al.* 2005; Bardgett and Wardle 2010; Kardol *et al.* 2013).

In restoration ecology, a common response to increased threat from invasive species is to implement management strategies to reduce the prevalence and spread of the incoming plant species (Matthews and Spyreas 2010); however, in the context of wetland restoration the act of creating or rehabilitating an area to meet hydrologic standards and goals may open that ecosystem to increased invasion risks (Seabloom and van der Valk 2003a; Gleason *et al.* 2011; Moreno-Mateos *et al.* 2015; Schlatter *et al.* 2016). Legacies caused by a wetland restoration physical disturbances, such as hydrology alterations or creating new wetland area through soil removal, also have the potential to alter plant–soil relationships and associated feedbacks on ecosystem processes (Suding *et al.* 2004; Kardol *et al.* 2013; Minnick and Alward 2015). Soil removal associated with wetland creation and restoration from non-wetland ecosystems have been shown to consistently alter soil biogeochemical processes, including reductions in soil organic material and microbial biomass (Bruland and Richardson 2005; Rokosch *et al.* 2009; Moreno-Mateos *et al.* 2012). These differences in soil processes are frequently long-standing and may have indirect impacts on the invasibility and overall plant diversity above-ground. Due to these important interactions in management goals, the invasive plant and soil feedbacks associated with restoration legacies are becomingly increasingly better understood (Klironomos 2002; Bardgett and Wardle 2010; Van der Putten *et al.* 2013). However, less research has focused on restoration and invasion plant–soil feedbacks in annual-dominated systems driven by high interannual variability despite the potential for strong plant controls on the long-term success of restoration practices and changes in ecosystem processes. Serving as a model system that fits these parameters, we sought to examine the interacting drivers of ecosystem change using a n annual dominated vernal pool system in California, USA that experiences high inter-annual variability.

**Figure 1.**
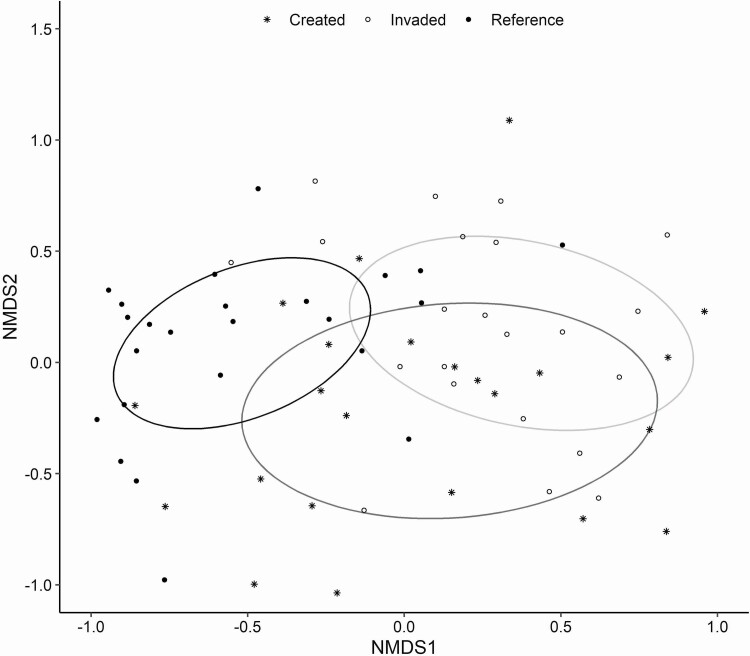
Non-metric multi-dimensional scaling ordination showing differences in community composition based on plant species abundance among pool types. Ellipses around the mean score for each pool type visually represent communities that are more or less similar, points and ellipses closer together indicate more similar composition. The ellipse for the created pools are grey, invaded pools are light grey and the reference pools are black. The 2D ordination solution had 0.20 final stress.

Vernal pools are shallow ephemeral wetlands found in flat to low slope grasslands with poorly draining soil that facilitates ponding. They are predominately precipitation-fed and defined by abrupt edges delimited by locations of ponding (Keeley and Zedler 1998). Historically they were able to avoid invasion because of the highly dynamic annual ponding cycle (Gerhardt and Collinge 2003, 2007; Faist and Beals 2018); however, recent extreme climate events, conversion to agricultural land and habitat disturbance have reduced habitat availability and allowed invasive encroachment into the pool boundaries (Pyke 2004; Collinge *et al.* 2011, 2013; Tanentzap *et al.* 2014; Schlatter *et al.* 2016; **see****Supporting Information—Fig. 1**). Future warming in these systems will promote increased evaporative losses thereby shortening inundation periods and promoting invasion by non-native plant species (Montrone *et al.* 2019). This recent change in abundance of native and non-native invasive species, combined with a need for active restoration in this ecosystem type makes it an ideal study system to address research questions focused on the implications of invasion on community contributions to ecosystem function and the comparative legacy effects associated with creation of new wetlands as part of restoration practices (Moreno-Mateos *et al.* 2012).

To examine the consequences of invasion in the context of restoration through plant–soil feedbacks on ecosystem processes, we examined a series of natural reference pools dominated by native species, naturally occurring pools invaded by non-native plants and created vernal pools dominated by non-native plants all located in a grassland site in central California. Among these contrasting restoration and invasion legacies we asked three primary questions: (i) How does invasion and restoration in vernal pool systems influence above-ground litter decomposition? (ii) How might shifts in above-ground community composition impact litter inputs to soils? (iii) Have shifts in above-ground community composition prompted changes in below-ground ecosystem function? Within this framework we hypothesize that ecosystem processes being driven by the above-ground presence of invasive plants will increase both carbon and nitrogen into the soil through altered plant litter and decomposition rates causing positive feedbacks, yet the plant material chemical ratios will favour increased nitrogen and thus increase microbial biomass in the invaded sites regardless of restoration status. Alternatively, active restoration, such as creating a disturbance of scraping the topsoil layer, has the potential to expose new soil layers lowering overall carbon and nitrogen content and increasing C:N ratios, pH and altering soil texture thereby producing lasting effects below-ground that could potentially overshadow the impacts of biotic invasion.

## Methods

### Site characteristics and experimental design

We conducted our experiment in the Central Valley of Solano County, CA, USA (38°16′N, 121°58′W). This region experiences a strong seasonality, with most precipitation falling during the winter months between November and February, a mean annual temperature of 20.1 °C, and mean annual rainfall of 500 mm (Climate of Sacramento, Report 2010). In our study site located at the Travis Air Force Base, Solano County, CA, both naturally occurring ‘reference’ vernal pools, that have existed in a single grassland meadow for decades, as well as pools created for restoration purposes are present across the 15-ha study site. This meadow contains a network of pool basins and upland grassland vegetation representative of regional vernal pool and grassland systems (**see****Supporting Information—Fig. 1**; Collinge and Ray 2009). To examine the ecological implications of invasion in the context of restoration, we focused on comparisons between created (artificially built pools for restoration purposes) and naturally occurring vernal pools all located in the same geographic area. The ‘created’ pools were created in 1999 to mimic the physical dimensions of the nearby reference pools (Collinge and Ray 2009) through manually digging out the basins by removing ~20 cm of topsoil. All created pools received a seeding treatment (Collinge and Ray 2009), which initially demonstrated a high instance of native species; however, this above-ground signature is no longer present and while natives are present in a subset, all created pools are now dominated by invasive species (**see****Supporting Information—Fig. 1**; Collinge *et al.* 2013; Faist and Collinge 2015; Faist and Beals 2018).

Over time (approximately in years 2006–08), a subset of the naturally occurring pools in this landscape also became dominated by invasive non-native annual grasses (hereafter ‘invasive’) while other pools eluded invasion, maintaining native species (**see****Supporting Information—Fig. 1**; Gerhardt and Collinge 2007; Collinge *et al.* 2013; Faist and Collinge 2015; Faist and Beals 2018). Given these delineations across invasion levels we identified three distinct pool types associated with their above-ground vegetation and restoration legacy with pool creation within the larger site context: (i) naturally occurring reference pools dominated by native species and the most desirable pool type (henceforth reference), (ii) naturally occurring reference pools dominated by invasive species (henceforth invaded) and (iii) pools created for restoration, which have been overtaken by invasive species (henceforth created). Importantly, there are no created pools containing only native species and thus a fourth category was not possible. We focused on 24 total pools for this study (Faist and Collinge 2015; Faist and Beals 2018), including created pools (5 × 20 m in size) located 10–100 m from reference and invaded pools to avoid pool spatial clustering across the field. To incorporate ecosystem functional differences associated with an inundation gradient, and to account for differences in the duration of hydric soils, from a pool edge to pool bottom we also sampled three general vegetation bands located within each individual pool associated with differences in inundation depths and durations (Emery *et al.* 2009; Russell and Beauchamp 2017). While there is variation within the vegetation zones, these three primary zones (or ‘locations’) include: (i) pool bottom, (ii) transition and (iii) edge zones (Emery *et al.* 2009). These zones experience differences in environmental drivers of key ecosystem processes, including shifts in inundation levels and therefore timing of soil aerobic versus anaerobic conditions, potential for light and UV interception for decomposing plant litter (Austin and Vivanco 2006; Austin *et al.* 2016) and potential soil properties such as texture associated with sediment settling (Javornik and Collinge 2016). We conducted an above-ground plant species composition survey in 2011 during peak biomass (see Faist and Beals 2018 for detailed description) that sampled each elevational band within the three vernal pool types (invaded, reference created; total 24 pools for 72 surveys). Species counts were used to scale plant-chemistry traits from individual species and their invasion status to multivariate community chemical trait matrices, including nitrogen (N), carbon (C), lignin (L) and cellulose.

### Plant chemistry

We also assessed variation in the chemistry of plant tissues from 18 vernal pool species as well as upland dominant species common to the site and surrounding grassland communities, to examine community-scale changes in stoichiometry and tissue chemistry from individual plants distributed across the site. These metrics were selected to target potential plant inputs on nutrient and carbon cycling among the three different vernal pool types. Specifically, we measured plant tissue concentrations of nitrogen (N), cellulose, lignin and carbon (C), and calculated the associated ratios between these components carbon:nitrogen (C:N) and lignin:nitrogen (L:N). All plant species were collected during peak biomass in 2011 coinciding with species composition surveys as measurement of growing season standing pools of plant chemistry. Cellulose and lignin, which are important components in litter decomposition and photodegradation (Austin *et al.* 2016), were determined by EcoCore Analytical services at Colorado State University, Colorado, USA, using a modified Goering–Van Soest forage fibre technique (Goering and Van Soest 1970). Plant C and N metrics were measured at the University of Colorado, USA, in N. Barger’s lab using a combustion technique (ECS 4010 CHNSO Analyser, Costech Analytical Technologies, Valencia, CA, USA).

### Vegetation decomposition

To reduce biomass accumulation, as this grassland site is ungrazed by herbivores, the site is mowed annually in the spring growing season (A. M. Faist, personal conversation); thus, we used recently cut foliage, collected from both upland and pool areas across the larger research site, for key species during spring peak biomass the season prior to field implementation to determine *in situ* above-ground plant decomposition rates through the utilization of litter bags. While using vegetation from peak biomass prevents the potential for nutrient translocation and resorption and consequently influences tissue chemistry, plant inputs in this system do not commonly undergo full senescence prior to mowing and deposition on the landscape, similar to Schuster *et al.* (2017). All litter bag plant material was clipped 2 cm above-ground level, and then dried at 60 °C until mass was stable (~72 h). Litter was clipped to a uniform size and placed into 10 × 10 cm litter bags (0.8 mm mesh) with initial weights recorded. Litter bags of each species were then placed in the replicate pool types (reference, invaded, created; total 24 pools) at three elevational locations within each pool (bottom, transition and edge). To account for effects of surrounding naturally occurring litter, as well as photodegradation, we placed equal replicates of litter bags on top of 5 cm of field-collected invasive grass litter and below 5 cm of the same litter type at the soil surface, hereafter referred to as ‘above’ or ‘below’ litter (*N* = 72 litter bags above field-collected invasive litter and *N* = 72 litter bags below). While 1 cm of litter has been shown to impact vernal pool vegetation structure (Faist and Beals 2018), placing bags underneath ~5 cm depth is sufficiently deep enough to avoid potential effects on decomposition from slight changes in depth due to wind or other disturbances.

To examine environmental and biotic drivers of vernal pool litter decomposition we installed litter bags for two winter wet seasons during the period when ponding is expected and one summer dry season, as well as comparing species effects on decomposition for the first wet season. We installed ‘wet season’ litter bags in the fall prior to any substantial precipitation (2011–12 and 2012–13) and removed them in the spring after pools dried down and the annual vegetation had bolted. These field incubations for the winter wet decomposition studies lasted from September to April. We placed the ‘dry season’ litter bags (2013) in the field just after spring drydown and removed them before any substantial autumn rains (April–September).

For the first year of the study, we were able to include litter bags for both the dominant native and invasive species for the wet season 2011–12 sampling period to test for species-level non-native invasive versus native decomposition differences. The invasive species litter was composed of pure *Lolium multiflorum*, an non-native invasive annual grass species that is ubiquitous in both created and invaded reference pools at the study site (Gerhardt and Collinge 2007; Collinge *et al.* 2011; Faist *et al.* 2013; Faist and Collinge 2015; Faist and Beals 2018). The native litter used was a vernal pool-adapted native annual grass, *Pleuopogon californicus*. While invasive and native species were used for the initial wet season litter bag study (2011–12), only the invasive grass *L. multiflorum* was used in the sequential litter bag deployments (wet season 2012–13 and dry season 2013) due to low site-level abundance of the native grass *P. californicus*.

### Soil properties

We collected bulk soil samples during peak flowering and peak above-ground plant biomass (April) in 2011 as the annual plants are dependent on soil processes while active during this brief period of highest productivity. Three soil samples were collected per pool, with one sample at each of three elevational plot locations within each pool (bottom, transition and edge). Soil samples (*N* = 72 at 125 cm^3^ each) were placed on dry ice immediately upon sampling. Samples were kept cold at 4 °C until processed, with the majority of soil analyses (microbial biomass, soil moisture, pH, C:N) completed within 1 month of field collection (all soil analysis associated with a single metric were processed at the same time).

We obtained soil moisture through the gravimetric soil moisture method (% soil moisture = 100 * (fresh weight − dry weight)/dry weight). We measured soil pH using a 1:3 soil to water ratio (Beckman pH/Temp meter model #340, Abbott Laboratories, Waukegan, IL, USA) on soil subsamples, where the pH subsample was measured three times to calculate a mean value for each elevational location within each pool. We used a subset of the pool bottom soils to obtain soil texture (percent sand, silt and clay; total 24 samples) using a modified version of Kettler *et al.* (2001) protocols for a rapid soil texture analysis. We selected the pool bottom samples specifically, as they have the greatest influence on soil drainage potential in vernal pools (Javornik and Collinge 2016). We also measured percent soil C and N for a bulk soil subsample at each location within all pools using elemental analysis with a CHN analyser (ECS 4010 CHNSO Analyser, Costech Analytical Technologies, Valencia, CA, USA, Sheldrick 1986; total 72 samples). Finally, we used a subsample of collected soil from pool bottoms to measure carbon microbial biomass per gram of dry soil as a metric for biotic responses to non-native invasive species and restoration (total 24 samples). To obtain soil microbial biomass carbon, we used the chloroform extraction method as described in Jenkinson *et al.* (2004), all samples were run together for comparison across treatments.

### Statistical analysis

For all univariate data analysis, we used linear mixed-effects models in R version 4.0.0 (R Core Team 2020) in the lme4 package (Bates *et al.* 2015), and all models were then analysed using the Anova function from the ‘car’ package to detect statistical significances (Fox and Weisberg 2019) followed by multiple comparisons using the ‘emmeans’ package (Length 2020). Models were checked for assumptions of normality associated with data distributions and residual deviations from the model, and appropriate transformations applied as described below. For the analysis of litter decomposition data, our initial analysis included interactions between fixed effects of pool type, year and the placement of litter above or below the existing litter layer. This model included location within pool (bottom, edge, transition) as a random effect to account for within-pool variation in inundation. Due to the potential for interactions between years and our fixed effects we also conducted analyses on annual and seasonal subsets of data to more carefully examine potential interactions among drivers of decomposition. These models included only pool type and litter placement as fixed effects, with location within a pool as a random effect. For the 2011 wet season we also subset the analysis by litter species type, as this was the only occurrence there were two sources of litter (native vs. invasive), therefore creating separate models for each species type. For the abiotic soil metrics of C:N ratio, soil moisture and pH, we used linear mixed-effects models including location within a plot (bottom, transition, edge) as a random effect and pool type (created, invaded, reference) as the main fixed effect. Soil texture and biotic metric microbial biomass C were analysed using linear regression with pool as the main predictor followed by statistical significance tests and pairwise comparisons used the same techniques described for litter decomposition. Litter decomposition as percent mass loss and soil C:N values were log-transformed due to the distribution of ratio data (Isles 2020), and soil microbial biomass C values were log-transformed to meet assumptions of normality. Visualization of the microbial biomass C included examining the relative difference (or effect) from reference pools as a conservative comparison for changes in this metric (0 values indicate no change from reference pools) along with 95 % confidence intervals to indicate pool-type differences for the created and invaded pools.

For our multivariate analyses, we examined differences among pool types based on community-scale chemistry traits, including tissue C, N, lignin and cellulose. Community composition was used to scale chemistry traits based on abundance of dominant plant species present in vegetation plots during the same year as tissue sample collection for chemical analysis (2011). We created species traits matrices based on multiplying species counts by species-level chemical traits, thereby creating four independent species trait matrices scaled by species presence, in addition to the community composition matrix of species counts that included all species present in each plot. These trait and composition matrices were examined for differences among pool types using a permutational multivariate analysis of variance (perMANOVA) test based on a Bray–Curtis dissimilarity matrix (R package Adonis). Multiple comparisons among pool types were conducted using the function pairwiseAdonis, which accounts for multiple comparisons and uses a Bonferroni correction (Martinez Arbizu 2020). We applied a relativization by maximum to all multivariate species data prior to creating the species-level distance matrix used for running the perMANOVA.

## Results

### Plant community chemistry

Given a known shift in the composition of the above-ground community associated with invasion into natural and created vernal pool types (Collinge *et al.* 2011; Faist and Beals 2018) we were interested in comparing potential shifts in community-scale vegetation chemistry due to changes in dominance of native and invasive species. We found substantial variation in species-level chemistry across commonly occurring species found at the site. This was particularly true between invasive and native species in the same growth form such that invasive non-native legumes had twice the concentration of N in above-ground tissue as native forbs (Table 1). In contrast, invasive non-native grasses, on average, had less than 50 % as much N as compared to the dominant native grass present at the site. Additionally, we found that both invasive non-native grasses and forbs had higher lignin concentrations than native species promoting a potential decrease in plant material decomposability associated with shifts in dominance between invasive and native. At the community scale we found that plant community composition of vernal pools was predicted by the type of pool in our study (*F*_2, 69_ = 4.9, *P* < 0.01) with different compositions in reference pools from pools created through creation (*F* = 6.7, *P* < 0.01) or affected by invasive species (*F* = 6.3, *P* < 0.01; Fig. 1). These shifts in composition translated into changes in plant community chemistry as scaled by counts of individual species, such that C concentrations (*F*_2, 69_ = 4.9, *P* < 0.01), N (*F*_2, 69_ = 4.9, *P* < 0.01), lignin (*F*_2, 69_ = 4.9, *P* < 0.01) and cellulose (*F*_2, 69_ = 4.9, *P* < 0.01; Table 2) community traits were altered, with the reference pools contributing different community plant chemical traits than either the created pools dominated by invasive species, or invaded pools (Fig. 1; Table 2).

**Table 1.** Plant tissue chemistry for common species among vernal pool types. *Species used in plant material decomposition experiment.

Functional group	Native status	Scientific name	N	C	Cellulose	Lignin	C:N ratio	L:N ratio
Forb	Invasive	*Convolvulus arvensis*	2.9	43.2	23.8	11.4	15.2	4.0
		*Erodium botrys*	1.0	41.9	32.7	7.1	42.2	7.1
		*Lotus* sp.	4.9	43.9	28.6	8.8	9.0	1.8
		*Rumex* sp.	2.8	44.9	30.4	4.4	15.8	1.5
		*Sonchus asper*	1.5	40.0	28.1	10.6	27.4	7.3
		*Vicia villosa*	4.4	43.7	28.1	14.9	9.9	3.4
	Native	*Achyrachaena mollis*	1.2	40.8	30.1	6.1	34.3	5.1
		*Eryngium vaseyi*	1.9	41.8	26.8	3.5	22.1	1.8
		*Lasthenia conjugens*	2.2	43.5	28.5	9.8	19.	4.4
		*Layia chrysanthemoides*	1.9	43.8	30.3	6.2	23.7	3.3
Grass	Invasive	*Avena fatua*	0.6	43.8	28.7	6.4	74.9	10.9
		*Bromus diandrus*	0.7	41.9	32.4	9.9	59.6	14.1
		*Bromus hordeaceus*	0.8	42.3	38.5	8.1	53.5	10.3
		*Hordeum marinum*	1.0	43.0	33.0	3.4	43.3	3.4
		Lolium multiflorum*	**1.3**	**42.5**	**32.8**	**5.9**	**34.0**	**4.7**
		*Polypogon maritimus*	1.9	44.0	33.5	9.3	23.5	4.9
		*Taeniatherum caput-medusae*	1.4	41.9	41.4	9.1	29.8	6.5
	Native	Pleuropogon californicus*	**2.6**	**42.1**	**27.4**	**4.0**	**16.3**	**1.5**

**Table 2.** Differences in multivariate plant community chemistry traits among pool types.

Traits	Pool-type comparisons	*F*	Adjusted *P*-value
Carbon	Invaded vs. Reference	6.32	<0.01
	Invaded vs. Created	1.80	0.27
	Reference vs. Created	6.66	0.01
Nitrogen	Invaded vs. Reference	6.32	<0.01
	Invaded vs. Created	1.80	0.34
	Reference vs. Created	6.66	<0.01
Cellulose	Invaded vs. Reference	6.32	<0.01
	Invaded vs. Created	1.80	0.31
	Reference vs. Created	6.66	0.01
Lignin	Invaded vs. Reference	6.32	<0.01
	Invaded vs. Created	0.04	0.33
	Reference vs. Created	6.66	<0.01

### Vegetation decomposition

**Figure 2.**
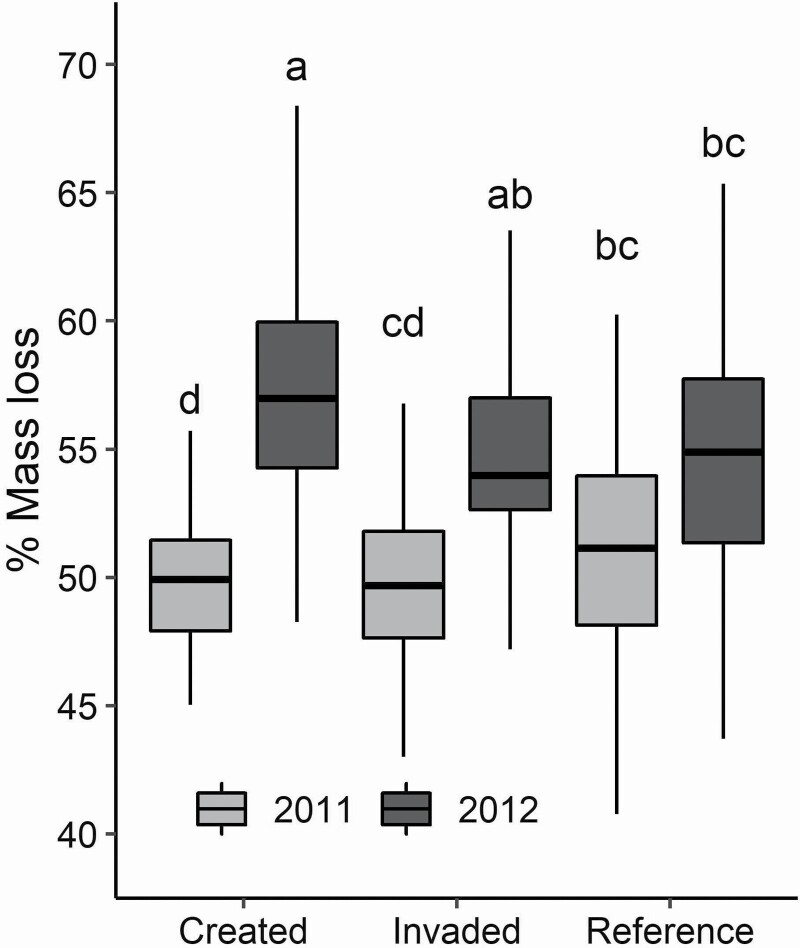
Invasive *L. multiflorum* decomposition percent mass lost by pool type for both winter wet seasons (2011–12 is noted as 2011 and 2012–13 noted as 2012). Same letters denote non-significant differences among pool types and years with an alpha of 0.05.

**Figure 3.**
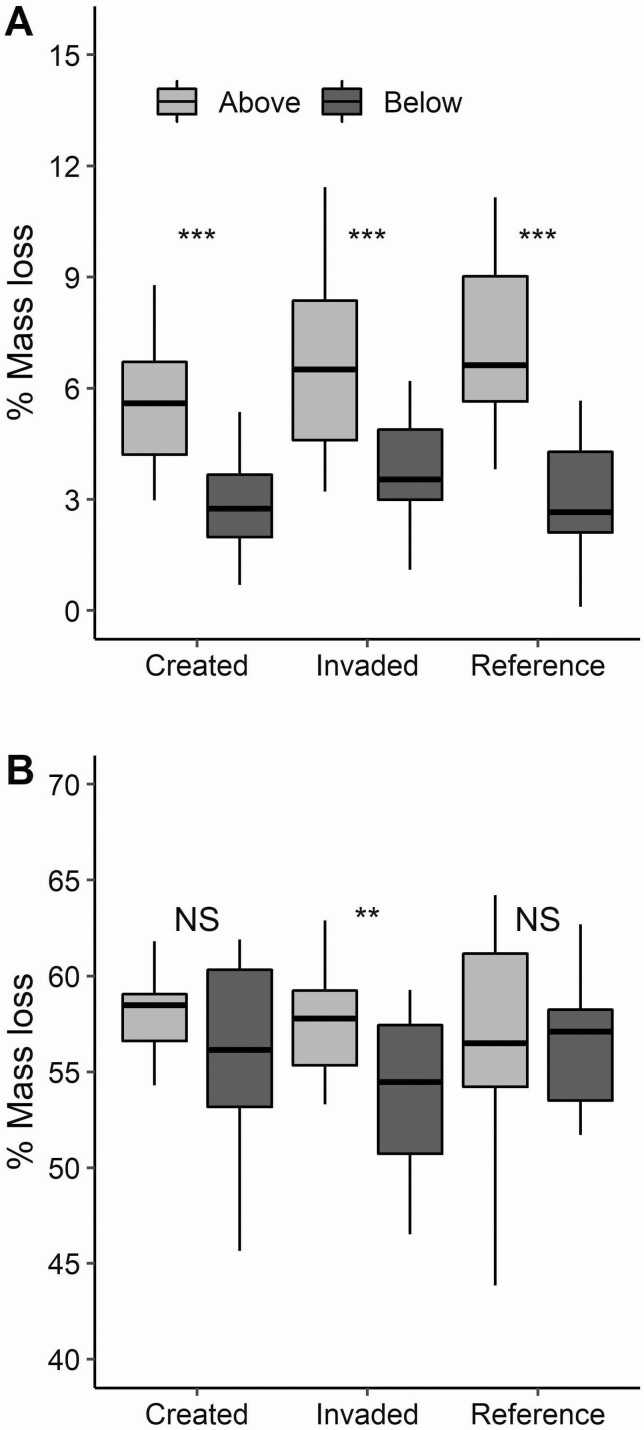
Decomposition of litter samples left in the field (A) for invasive *L. multiflorum* grass litter above and below the existing litter layer during the summer dry season of 2013 and (B) for native *P. californicus* grass litter and above and below the existing litter layer during winter of 2011. Same letters denote non-significant differences among pool types with an alpha of 0.05.

California vernal pools are highly dynamic and dependent on regional weather patterns creating ponding in the winter and desiccating soils in the summer, and we found that year and season had strong effects on above-ground plant material decomposition rates. Specifically, we found that litter bags placed during the winter wet season, comprised of invasive grass, decomposed nearly 10 times faster than the associated summer dry season litter bags (Figs 2 and 3). When comparing across winter periods, decomposition of invasive plant material was driven primarily by year (Fig. 2; Table 3) and to a lesser extent by pool type, likely associated with shifts in soil moisture and water table height. In particular, the created pools showed the greatest difference in decomposition between years followed by the invaded pools, while the reference pools did not vary. The comparatively more wet 2012–13 growing season (Faist and Beals 2018) increased overall decomposition by 5 % over invasive species litter bags in the drier year 2011–12. Additionally, the wetter year (2012–13) resulted in greater variation in decomposition associated with pool types. In contrast, decomposition of invasive litter during summer months was driven primarily by the placement of litter bags relative to the existing litter layer and no significant differences among pool types (Fig. 3A; Table 3).

**Table 3.** Statistical comparisons of the effect of pool type (PT; Restored, Invaded, Reference) on litter decomposition (% mass loss) for invasive and native plant litter during winter and summer seasons. *All % mass loss data were log-transformed for statistical analysis.

Response	Predictors	*F*-statistic	*P*-value	*R* ^2^m	*R* ^2^c
Invasive litter decomposition* (winter)	PoolType	*F*_2, 227_ = 2.2	0.11	0.30	0.30
	Year	*F*_1, 227_ = 83.3	<0.01		
	PT × Year	*F*_2, 226_ = 5.9	<0.01		
Invasive litter decomposition* (summer)	PoolType	*F*_2, 134_ = 2.8	0.07	0.40	0.41
	Placement	*F*_1, 134_ = 85.0	<0.01		
	PT × Placement	*F*_2, 134_ = 2.6	0.07		
Native litter decomposition* (winter)	PoolType	*F*_2, 120_ = 0.4	0.64	0.07	0.08
	Placement	*F*_1, 119_ = 4.9	0.03		
	PT × Placement	*F*_2, 119_ = 1.6	0.20		

Finally, decomposition differed substantially associated with species selection (*F*_1, 213_ = 91.6, *P* < 0.01; Figs 2 and 3B). We found that the native grass *P. californicus* decomposed significantly more than invasive grass, *L. multiflorum*, during the 2011–12 winter growing season they were in place. This may be linked to differences in species chemistry as the native species had higher N and lower lignin and cellulose than the invasive species (Table 1; bolded species). Decomposition of native litter during the winter months was not influenced by pool type, with little of the variation in mass loss described by either pool type or little bag placement (Table 3). Litter bag placement above existing litter layer and thus exposed to the sunlight differentially influenced the mass loss of native and invasive species plant material during the 2011–12 wet season. Native litter lost more mass when placed above the existing litter layer (Fig. 3B) while the invasive litter did not respond to placement (*F*_1, 92_ = 0.85, *P* = 0.36), suggesting differences in the drivers of decomposition linked with invasion.

### Soil properties

**Figure 4.**
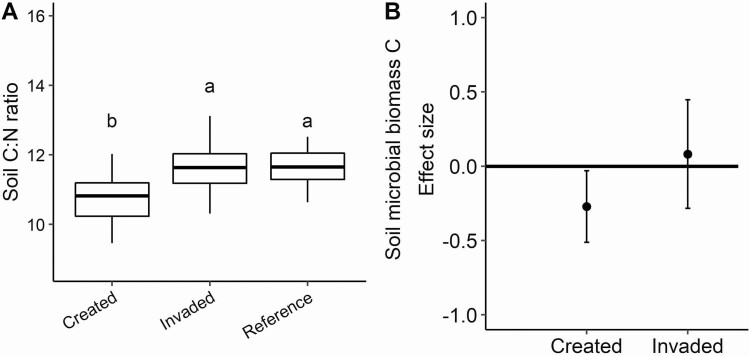
Differences in organic soil properties among pool types including (A) bulk soil carbon (C) to nitrogen (N) ratios and (B) soil microbial biomass. Boxplot and lettering follow the design in Fig. 3.

Many of the fundamental physical soil properties we measured did not differ among vernal pool types associated with invasion or creation (Table 4); however, there was some variation especially for organically derived measurements (Fig. 4). Soil moisture collected during peak flowering in the growing season averaged 16 %, with a significant effect of pool type (Table 4), such that invaded pools had higher soil moisture than created pools. Invaded pools also demonstrated a greater variation in moisture levels than either the reference or created pools. Soil pH also varied among pool types (Table 4) with invaded pools having lower pH than reference pools. In comparison, the soil texture was strikingly similar across pool types with a notably high silt content (~56–60 %) and low sand content (~19 %; Table 4). We observed strong differences in the soil C:N ratio among pool types (Fig. 4A; *F*_2, 68_ = 11.9, *P* < 0.001, *R*^2^m = 0.23, *R*^2^c = 0.30) where the created pools (C:N-10.7) were significantly lower than the reference (C:N-11.7) and invaded pools (C:N-11.8). We found that soil microbial biomass C when compared across pool types also varied (Fig. 4B; *F*_2, 21_ = 4.6, *P* = 0.02, *R*^2^ = 0.28). The lowest average microbial biomass was found in the created pools and was significantly lower than the reference pools based on effect size.

**Table 4.** Observed physical soil properties for the different pool types. Same letter designations indicate non-significant differences among pool types for each soil property from linear mixed-effects models with pool type as the fixed effect and location as a random effect.

			Naturally occurring			
		Created	Invaded	Reference	*F*-statistic	*P*-value
Soil texture (%)	Clay	24.5 ± 3.3a	24.8 ± 3.8a	21.7 ± 0.9a	*F*_2, 21_ = 0.34	0.71
	Silt	56.6 ± 2.6a	56.1 ± 1.9a	59.6 ± 2.4a	*F*_2, 21_ = 0.65	0.53
	Sand	18.9 ± 3.1a	19.1 ± 2.9a	18.8 ± 2.5a	*F*_2, 21_ = 0.004	1
Soil moisture (%)		13.4 ± 0.7b	18.6 ± 1.6a	15.3 ± 1.3ab	*F*_2, 67_ = 4.37	0.02
Soil pH		5.9 ± 0.7ab	5.6 ± 1.6b	5.9 ± 1.3a	*F*_2, 66_ = 4.34	0.02

## Discussion

The introduction of invasive species can alter the ecosystem through a variety of pathways creating amplifying feedbacks that exacerbate further ecosystem changes (Simberloff and Von Holle 1999; Suding *et al.* 2004; Faist and Beals 2018). To combat invasion, ecological restoration efforts are often put in place to enhance non-invasive native establishment and improve ecosystem health. Many restoration actions include altering the soil surface in some capacity, often proportional to the degree of intervention required for a project, and these modifications can further influence potential plant–soil feedbacks associated with invasive species (Eviner and Hawkes 2008). To better understand the legacy of active restoration and invasive plant establishment, we examined the impacts of physical restoration efforts and non-native invasive plant impacts on vernal pool plant–soil properties above- and below-ground. Our study found that invasive species in this system, due to their plant tissue chemistry, demonstrated or had the potential for slower rates of litter decomposition than native species, with decomposition of invasive non-native grass litter driven by season and changes in interannual precipitation. These findings suggest that above-ground community structure shifts to favour invasive dominated species has the potential to impact plant inputs to below-ground processes over long-term shifts in plant inputs to soils. While the potential was there, instead, we found that abiotic soil legacies resulting from the vernal pool restoration excavation process more than 10 years prior were stronger drivers of soil functions, with restored pools containing lower soil C:N ratios, and trends in lower microbial biomass. Importantly, the existing disconnect between above-ground invasion and below-ground function suggests that restoration practices aimed at controlling community structure may be sufficient for promoting long-term success; however, additional efforts may be appropriate to account for legacy effects of restoration on below-ground processes.

### Litter decomposition and plant community chemistry

Invasive species can alter ecosystems through a wide variety of pathways, ranging from shifts in community interactions to changes in nutrient cycling and physical ecosystem properties (Ehrenfeld *et al.* 2005; Hobbie 2015) and this has been found in a number of wetland systems (Angeloni *et al.* 2006; Fickbohm and Zhu 2006; Liao *et al.* 2008). In our study, vernal pool communities were invaded by non-native annual grasses ~5–8 years prior to our study (**see****Supporting Information—Fig. 1**; Collinge *et al.* 2011, 2013). These invasive grasses possess fundamentally different traits than the smaller stature annual native community and we found evidence for shifts in community composition, community plant chemistry and litter decomposition associated with invasion. These shifts in community-level plant traits have implications for altering plant–soil feedbacks that are dependent on continued and future climate changes that impact hydrologic dynamics in vernal pool and wetland systems (Purcell *et al.* 2019). Numerous studies have found differences in decomposition rates between native non-invasive and non-native invasive species; however, many show the opposite trend to ours, with invasive species decomposing at a faster rate than their associated native species (Allison and Vitousek 2004; Liao *et al.* 2008; Bardgett and Wardle 2010; Spirito *et al.* 2014; Huangfu *et al.* 2019). This paradigm enables an amplifying feedback for the invasive species as faster decomposition can release more nutrients into the soil to further maintain an invasive presence (Ehrenfeld 2003; Allison and Vitousek 2004; Ehrenfeld *et al.* 2005; Liao *et al.* 2008). Research focused in wetland communities in the Eastern USA invaded by *Phragmites australis*, however, shows a similar decrease in decomposition rates driven by higher C:N ratios and higher concentrations of lignin ultimately promoting nutrient competition for native plant species (Windham and Ehrenfeld 2003).

While we observed that invasive non-native decomposition was slower than native species in our study, an alternate positive feedback to soil nutrients associated with invasive effect on the ecosystem has been noted in this vernal pool system (Faist and Beals 2018). Here the invasive litter layer creates a physical barrier, benefiting the non-natives while hindering the germination of small native annual species thus facilitating further invasion (Wolkovich *et al.* 2009; Faist and Beals 2018). Additionally, this accumulation of litter appears to reduce decomposition of existing litter, as our litter bags installed beneath the litter surface decomposed much more slowly than bags on top of existing litter during the dry season. This phenomenon of litter layers has been observed in other terrestrial systems and is a common above-ground amplifying feedback to invasion (Ehrenfeld *et al.* 2005).

Long-term averages of litter decomposition are driven largely by climate and microbial biomass (Bradford *et al.* 2017); however, at a local scale the tissue chemistry has been shown to have a substantial effect on field decomposition rates both for above-ground and below-ground litter components (Aerts 1997; Bradford *et al.* 2016; Huangfu *et al.* 2019). The higher the cellulose, overall carbon and/or lignin content, and lower nitrogen content within a plant, the field decomposition rates would be predicted to be slower than plant species with low cellulose and carbon and lignin contents (Cornwell *et al.* 2008). These observations held true in comparing trait differences between native forbs and invasive grasses at our site (Table 1) and the associated decomposition rates of selected target species. It must be noted that all plant tissue samples were collected during peak biomass and not after senescence allowing for nutrient translocation (Vergutz *et al.* 2012; Wang *et al.* 2018). This, however, is representative of the site-level management where annual mowing it conducted dropping the cut biomass directly on to the soils surface. Nonetheless, the decomposition values, or mass lost, presented here also serve as comparisons across the distinct species, pool types, years and seasons rather providing discrete decomposition rates.

Restoration ecology focused on evaluating restoration success in depressional wetlands more generally has suggested that the nutrient composition of vegetation is an important metric in evaluating long-term restoration success (Whigham *et al.* 2002). Shifts in community traits with restoration and invasion therefore imply fundamental deviations in the ecosystem from the intended targets, likely associated with long-term patterns of altered water availability (Montrone *et al.* 2019) that have cascading consequences on plant and soil processes. While litter source had an influence on litter decomposition rates, pool type did not impact the decomposition rate of said litter except during the dry season. This finding suggests that drivers of litter decomposition, such as differences in sediments or microbial processing, that may be unique to individual pool types have not yet diverged, despite other measured differences in ecosystem function. On the other hand, future changes in the duration of pool inundation and vegetation zone location or width associated with ongoing shifts from climate change may alter these drivers of decomposition unequally among the native versus invaded pools, ultimately promoting changes in decomposition rates that feedback to nutrient cycling in the ecosystem (Seabloom and van der Valk 2003b).

### Soil properties

Differences in plant community composition, tissue chemistry and decomposition rates can have a cascade of effects on the below-ground, such as speed of nutrient cycling, microbial community composition (Kourtev *et al.* 2002), as well as shifts in oxygen availability in the rooting zone and quality of below-ground plant-derived C (Leicht-Young *et al.* 2009; Maietta *et al.* 2020). Despite the potential for these changes associated with a novel plant species invasion and observed above-ground differences, we instead found a trend for lower microbial biomass and lower soil C:N ratios in the created pools as compared to the naturally occurring pools, whether invaded or reference. These findings are in agreement with many studies looking at long-term legacy effects of created restoration projects on the accumulation of soil carbon (Ballantine *et al.* 2012; Moreno-Mateos *et al.* 2012). Indeed, the nature of restoration for vernal pools often necessitates topsoil excavation and consequently the created pools—created in 1999—are much younger in their soil surface development and the carbon cycling may not have had sufficient time to match those of the reference pools as the accumulation of soil organic material associated with anaerobic conditions takes time (Knops and Tilman 2000; Hogan *et al.* 2004). Additionally, the potential for created or restored wetlands to sequester C in soils is frequently cited as a benefit to the restoration process generally (Gleason *et al.* 2011) and therefore it is not unexpected that our created pools had less C. The same restoration influence on C:N ratios would influence microbial biomass as less soil carbon supports fewer microbes, regardless of invasion status, and similar findings are common in wetland restoration projects in comparison with reference wetland areas (Cleveland and Liptzin 2007; Rokosch *et al.* 2009; Prasse *et al.* 2015).

While a direct effect of a restoration legacy was observed below-ground in soil C:N and generally in extracted microbial biomass C, not all soil metrics tested demonstrated this artefact of restoration nor demonstrated a change solely due to invasion, in contrast to studies comparing reference and restored pools with greater differences in vegetation community structure (i.e. woody plants; Hogan *et al.* 2004). Indeed, soil texture was similar across the site and neither drove susceptibility to invasion or was affected by restoration actions or invasion. This finding is important, as it implies that either texture has converged among pool types over time associated with overland flow and sediment settling, or that removal of the upper soil layers during creation processes did not fundamentally alter the pool soil texture. As soil texture is often linked with resource availability and microbial processing, many studies have highlighted long-term legacy effects on resource availability and soil processes associated with created wetland restoration projects (Bruland and Richardson 2005; Prasse *et al.* 2015). Our one-time measurement of soil moisture at peak biomass, on the other hand, was lowest in the created and highest in the invaded; however, both pool types did not differ from reference pools, which are native-dominated. While the invaded pools contained the highest soil moisture following our prediction that an invasive layer will retain soil moisture, the restored pools were not different from either of the natural occurring pools. We propose that the lack of a strong invasion trend below-ground could be due to an interaction between the litter layer and the amount of time associated with water draw down in the vernal pools. The native-dominated reference pools often have the longest ponding cycle and are the slowest to drain (Faist and Beals 2018) yet demonstrated a low soil moisture at peak biomass. Their exposed bare soil allowed by the small stature natives, and lack of litter layer could allow for a faster evaporation and subsequent drydown than rates in other pool types with a litter layer, therefore also influencing the duration of anaerobic soil conditions and rates/forms of soil respiration (Gleason *et al.* 2011). Alternatively, created pools have a shorter ponding time (Faist and Beals 2018) but the deep invasive litter layer retaining soil moisture may have caused the lack of observed difference between the reference and created pools.

Similar interacting processes likely influenced our findings for differences in soil pH among pool types that were not connected to either invasion or restoration independently. Invasive plants have been shown to alter the soil pH, both increasing and decreasing depending on the invasion type and magnitude (Vilà *et al.* 2011). These changes in pH are often an indirect artefact of changes in litter chemistry and nutrient cycling rates (Ehrenfeld 2003), and we originally predicted that the invaded sites would differ from reference vernal pools based upon accumulated differences in plant litter contributions to the soil. While the consequence of plant invasion may have not yet occurred in our system, the multiple differences in soil metrics among pools types that fall outside a simple restoration or invasion paradigm illustrate the complexity of potential plant–soil direct and indirect effects as altered by restoration and invasion. The climate change conditions promoting shifts in inundation duration and invasibility risk generally at the site are also likely to impact soil processes differentially among pool types associated with the presence of invasive species. Further research into these interactions may clarify this potential interaction and how it controls vernal species composition and restoration efforts.

## Conclusions and Future Directions

In summary, despite strong changes in above-ground plant contributions the soil metrics directly linked to plant inputs did not show the same response to the plant invasion. Instead, organic soil components exhibited a legacy of vernal pool active restoration from the decade prior. This study demonstrates that while above-ground vegetation may vary strongly in its abundance and composition and produce conditions that facilitate further invasion, this signal does not necessarily translate below-ground even after multiple years of invasion. The success of a restoration project is often based solely on the above-ground vegetation, and in this regard, further restoration efforts to minimize invasive species presence is required for our vernal pools. However, this study illustrates an opportunity to limit long-term effects of altered above-ground plant–soil feedbacks on below-ground processes should additional restoration occur. The importance of this time lag in establishing altered community feedbacks within the ecosystem provides a window of opportunity for adaptive management practices aimed at ecosystem recovery.

## Supporting Information

The following additional information is available in the online version of this article—

**Figure S1.** Site history and succession of vernal pools at the Travis Airforce Base in the Central Valley of California, USA, highlighting the formation of and changes to vernal pool types. Severe flooding on site occurred during wet season of 2006, which was followed by an extreme drought during the growing season of 2007 thereby facilitating a shift in above-ground plant composition (invasive species indicated using orange/brown coloration in figure). See references for details: Gerhardt and Collinge (2007); Collinge *et al.* (2011); Collinge *et al.* (2013); Javornik and Collinge (2016); Faist and Beals (2018).

plab042_suppl_Supplementary_Figure_S1

## Data Availability

All data associated with statistical analyses presented in this paper are available through Dryad (https://doi.org/10.5061/dryad.d2547d80g).

## Notes

**Plants, Ecosystems & Climate. Chief Editor:** Mary Heskel
