# Supplementary material for: Consequences of above-ground invasion by non-native plants into restored vernal pools do not prompt same changes in below-ground processes
Source: AoB Plants. 2021 Jul 5;13(6):plab042. doi: 10.1093/aobpla/plab042 (PMC8598383; doi:10.1093/aobpla/plab042)
Supplement: plab042_suppl_Supplementary_Figure_S1 [file plab042_suppl_supplementary_figure_s1.pdf]

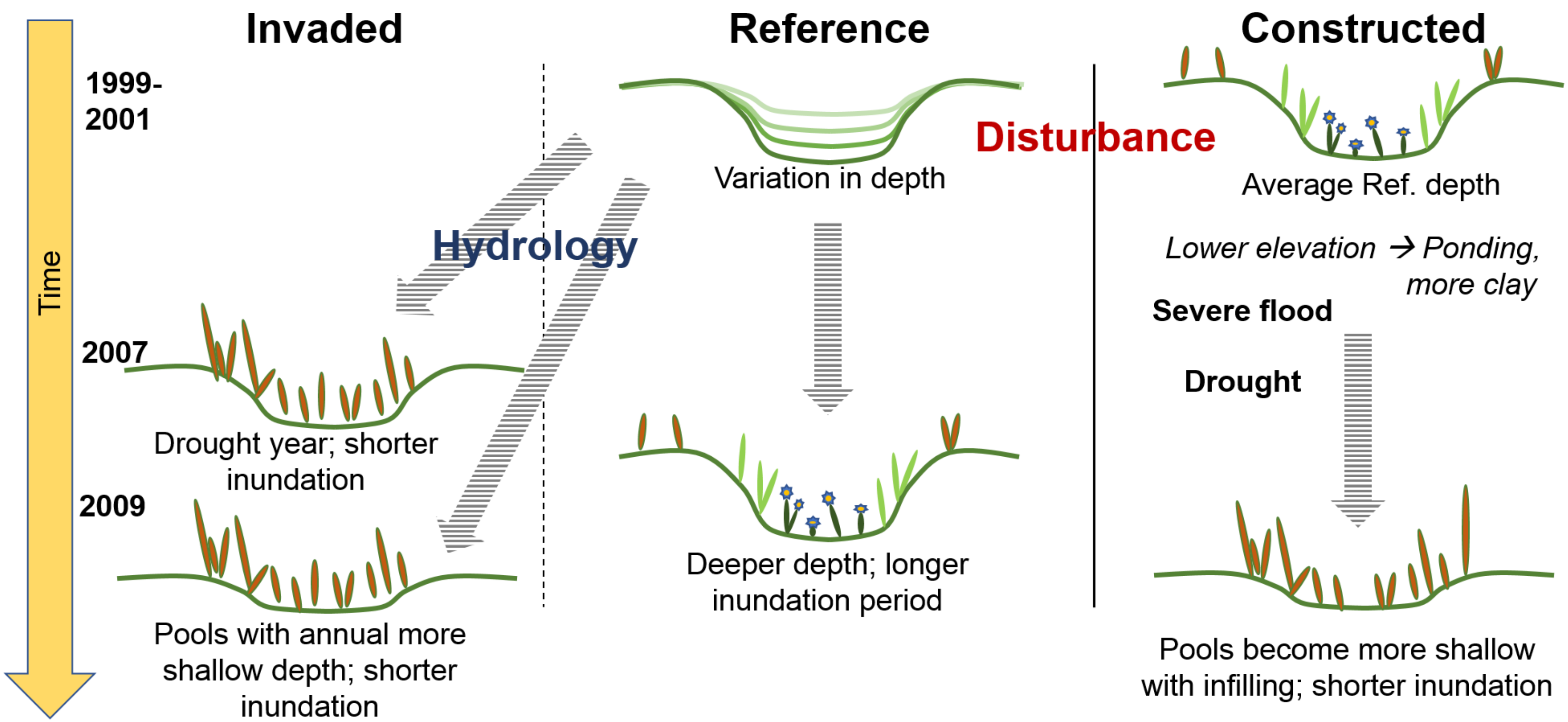

Supplemental Figure 1) Site history and succession of vernal pools at the Travis Airforce Base in the Central Valley of California, USA highlighting the formation of and changes to vernal pool types. Severe flooding on site occurred during wet season of 2006, which was followed by an extreme drought during the growing season of 2007 thereby facilitating a shift in aboveground plant composition (invasive species indicated using orange/brown coloration in figure). See references for details: Gerhardt and Collinge 2007, Collinge et al., 2011; Collinge et al., 2013; Javornik and Collinge 2016; Faist and Beals 2018.
